# The clinical characteristics, etiologic pathogens and the risk factors associated with dehydration status among under-five children hospitalized with acute diarrhea in Savannakhet Province, Lao PDR

**DOI:** 10.1371/journal.pone.0281650

**Published:** 2023-03-02

**Authors:** Latdavanh Vorlasane, Mai Ngoc Luu, Ranjit Tiwari, Atsuko Imoto, Miho Sato, Nguyen Tien Huy, Yasuhiko Kamiya, Kazuhiko Moji

**Affiliations:** 1 School of Tropical Medicine and Global Health, Nagasaki University, Nagasaki, Japan; 2 Department of Internal Medicine, University of Medicine and Pharmacy at Ho Chi Minh City, Ho Chi Minh City, Vietnam; 3 Online Research Club (https://www.onlineresearchclub.org/), Nagasaki, Japan; 4 B.P. Koirala Institute of Health Sciences, Dharan, Nepal; 5 School of Global Humanities and Social Sciences, Nagasaki University, Nagasaki, Japan; Regional Medical Research Centre Bhubaneswar, INDIA

## Abstract

**Background:**

Acute diarrhea is a common health problem in children, especially those under five years of age (U5). The mortality rate due to acute diarrhea among U5 children accounted for 11% in Lao PDR in 2016. No study has been done to investigate the etiologic pathogens of acute diarrhea and the risk factors associated with dehydration status among U5 children hospitalized with acute diarrhea in this region.

**Objectives:**

The study aimed to evaluate the clinical characteristics, etiological agents and associated factors of dehydration status of acute diarrhea among hospitalized U5 children in Savannakhet Province, Lao PDR.

**Methods:**

This retrospective study reviewed paper-based medical records with available stool examination results of 33 U5 children hospitalized with acute diarrhea in Savannakhet Provincial Hospital, Lao PDR between Jan. 2018 and Dec. 2019. Descriptive statistics were used to describe clinical characteristics and etiologic agents of acute diarrhea of the children. Nonparametric test, Pearson’s Chi-square test and Fisher exact test were used to determine the risk factors associated with level of dehydration of the participants.

**Results:**

Vomiting was the most common symptom (66.6%), followed by fever (60.6%). Dehydration was found in 48.4% of subjects. Rotavirus was the most common identified pathogen with a prevalence of 55.5%. Bacterial enteric infection was identified in 15.1% of patients. There is a significantly higher prevalence of dehydration among children with acute diarrhea caused by rotavirus compared to those with negative rotavirus testing (70.0% vs. 12.5%, p = 0.02).

**Conclusions:**

Rotavirus was the most prevalent pathogen of acute diarrhea among U5 children. Pediatric patients with acute diarrhea caused by rotavirus had a higher prevalence of dehydration compared to those with negative rotavirus testing.

## Introduction

Acute diarrhea is a common health problem in children, especially those under five years of age (U5). This condition contributes to significant morbidity and mortality in many low-income countries worldwide [1]. Despite an improving trend in mortality rates, acute diarrhea is still the leading cause of child death worldwide in the last decade, accounting for nearly 11% of deaths among U5 children and about 800,000 child deaths in developing countries annually [2].

In low-income countries, children who suffered from moderate-to-severe acute diarrhea were more likely to fail to thrive or die during follow-up [3]. In 2015, the U5 mortality rate of Lao People’s Democratic Republic (Lao PDR) was 53.6 per 1,000 live births, the highest in the Western Pacific region [4]. Up to 11% of U5 children deaths in Lao PDR were reported to be caused by acute diarrhea [5]. In Savannakhet Province—the largest province of Lao PDR, acute diarrhea is the primary disease that affects U5 children, with over 4,000 cases reported in 2018 [6, 7]. However, no study has been done to investigate the etiologic pathogens of acute diarrhea and the risk factors associated with dehydration status among U5 children hospitalized with acute diarrhea in this region.

In this study, we aimed to evaluate the clinical characteristics, etiological agents, and associated factors of the severity of acute diarrhea among hospitalized U5 children in Savannakhet Province, Lao PDR. We believe the results from this study can provide the evidence to guide the policy on management and prevention of acute diarrhea in U5 children in the country.

## Materials and methods

### Design and setting

The study was approved by the Ethical Committee of School of Tropical Medicine and Global Health, Nagasaki University, Japan (ethical code numbered NU_TMGH_2020_150_1) and the National Ethical Committee for Health Research, Lao PDR (ethical code numbered 085/NECHR). In this retrospective observational study, we reviewed hospital records and stool examination results of U5 children with acute diarrhea admitted to Savannakhet Provincial Hospital (SPH)–an urban government hospital in Lao PDR, between Jan 2018 to Dec 2019. During this study period, there were 3970 U5 children admitted (approximately 5 cases per day), of which gastrointestinal conditions accounted for 53.4% of admissions. Inclusion criteria included age of 0–59 months, defecation frequency of at least three times per day, loose or stools within 24 hours, diarrhea course within two weeks, and hospitalization due to diarrhea. The children who were hospitalized for other causes but not acute diarrhea or who did not have stool examination results were excluded. The Ethical Committees waived the informed consent of the participants because of the retrospective nature of the study.

### Data collection

Based on the papered-based medical records, demographic characteristics, including age, gender, and nutrition status at admission, were collected. In addition, data regarding clinical manifestations (fever, vomiting, bloody stool, days of diarrhea, severity of dehydration), treatment before and after admission, stool examination results, duration of hospitalization, and clinical outcomes at discharge were also recorded.

The nutrition status and the severity of dehydration were assessed according to the WHO guidelines by medical doctors on admission before management started. In SPH, all admitted pediatric patients were measured in weight and height for screening acute malnutrion (wasting) as routine only. The child with weight-for-height/length ≤ -2SD was classified as wasted [8]. The dehydration status in acute diarrhea was classified as no dehydration, some (moderate) dehydration, or severe dehydration based on the presence of lethargy/ unconsciousness, restlessness/ irritability, sunken eyes, ability to drink, and skin turgor [9]. The stool samples were collected within 24 hours of admission and stored in a freezer at 20°C. At the end of each day, all collected stool specimens were shipped on dry ice to the National Center Laboratory and Epidemiology (NCLE) and analyzed the following day. The pathogens that could be detected include *Salmonella*, *Shigella*, *Vibrio cholera*, *Vibrio parahaemolyticus*, *Aeromonas*, *Plesiomonas shigelloides*, *Campylobacter*, *Escherichia coli*, and Rotavirus.

### Statistical analysis

The collected data were organized in an Excel spreadsheet (Microsoft Corp., Redmond, Washington, USA) and then analyzed using Stata version 12.0 (StataCorp LLC, Texas, USA). Descriptive statistics were used to describe the demographic and clinical characteristics of the participants and the etiologic agents of acute diarrhea. We presented quantitative data with normal distribution as mean (standard deviation) and those without normal distribution as median (range). The Pearson chi-square or Fisher’s exact test was used to analyze categorical data. Normally-distributed continuous variables were compared using Student independent t-test, while the Mann-Whitney U tests were performed to compare those without normal distribution. A p-value less than 0.05 was considered statistically significant.

## Results

### Baseline and clinical characteristics

There was a total of 6764 U5 children with acute diarrhea visiting the Outpatient Department of SPH. Among them, 1895 cases were hospitalized, but only 33 patients had available hospital records with stool examination results. The socio-demographic characteristics of these 33 children are summarized in Table 1. Their median age was 12 months (range 0.2 to 48). 93.9% of cases were 0–35 months of age. The male/female ratio was 3.13/1. The information to assess the nutrition status was only available for twenty-five cases (75.7%), out of which five (20.0%) were malnourished.

**Table 1:** Demographic and clinical characteristics.

Characteristics	Total (n = 33)	No dehydration (n = 17)	Dehydration (n = 16)	p-value
**Median age** (months)	12 (0.2–48)	13 (3–36)	9 (0.2–48)	0.5***
**Age groups**				0.07*
Infants (0–11 months)	15 (45.4)	6 (35.3)	9 (56.2)	
Toddlers (12–35 months)	16 (48.5)	11 (64.7)	5 (31.3)	
Preschools (36–59 months)	2 (6.1)	0 (0.0)	2 (12.5)	
**Gender**				1.0*
Female	8 (24.2)	4 (23.5)	4 (25.0)	
Male	25 (75.8)	13 (76.5)	12 (75.0)	
**Malnutrition** ^#^	5 (20.0)	3 (23.1)	2 (16.6)	1.0*
**Vaccination status**				0.1*
Up-to-date	11 (33.4)	4 (23.6)	7 (43.7)	
Completed	18 (54.5)	12 (70.6)	6 (37.6)	
Not completed	14 (12.1)	1 (5.8)	3 (18.7)	
**Duration of illness before admission** (days)	2 (1–6)	2 (1–6)	2 (1–5)	0.4***
**Duration of diarrhea before admission** (days)	3.4 ± 1.8	3.4±1.8	3.4±1.8	0.9****
**Clinical manifestations on admission**				
Fever	20 (60.6)	13 (76.4)	7 (43.8)	0.055***
Vomiting	22 (66.7)	9 (52.9)	13 (81.2)	0.08***
Bloody stool	2 (6.0)	2 (11.8)	0 (0.0)	0.4*
**Received antibiotics before admission**				1.0*
Yes	9 (27.3)	5 (29.4)	4 (25.0)	
No	22 (66.7)	11 (64.7)	11 (68.8)	
Unknown	2 (6.0)	1 (5.9)	1 (6.2)	

^#^Only 25 cases had measured weight-for-height/length to assess for acute malnutrition status (wasting). Numbers in the parentheses indicate column percentage (%) unless indicated range for continuous variables.

*P-value was calculated by Fisher’s exact test.

**P-value was calculated by Chi-squared test.

***P-value was calculated by Mann-Whitney test.

****P-value was calculated by Student’s t test.

The mean duration from the onset of diarrhea to hospital admission was 3.4 ± 1.8 days. On admission, vomiting was the most common symptom accounting for 66.7% of patients, followed by fever (60.6%). The bloody stool was found only in two patients. Before admission, 27.3% of cases received antibiotics. As no severe dehydration was recorded, we divided the study individuals into two groups: 17 (51.5%) patients with no dehydration and 16 (48.5%) patients with dehydration (some dehydration). These two groups were not significantly different in their demographic and clinical characteristics (p>0.05).

### Etiologic agents of acute diarrhea in children under five years

Among the 33 cases, only 18 patients (54.5%) were tested for the presence of rotavirus in stool samples. Rotavirus was the most commonly identified pathogen, with a prevalence of 55.5% (10/18). Bacterial enteric infection was identified in 15.1% of patients (5/33). No patient had a bacterial co-infection. The rates of identified enteric pathogens are shown in Table 2. In two subjects presented with bloody stool, one had a positive *Shigella spp*. fecal test, and the other one suffered from acute diarrhea caused by *Salmonella spp*.

**Table 2:** Distribution of identified enteric pathogens in U5 children with acute diarrhea.

Etiologic agents	Number (%)
Rotavirus*	10 (55.5)
*Salmonella spp*.	4 (12.1)
*Shigella spp*.	1 (3.0)
*Vibrio cholera*	0 (0)
*E*. *coli*	0 (0)
*Campylobacter*	0 (0)
Others (*Vibrio parahaemolyticus*, *Aeromonas*, *Plesiomonas and shigelloides)*	0 (0)

*Only 18 cases were tested for rotavirus.

### Treatment and outcome of acute diarrhea

All subjects were treated with oral rehydration solution (ORS) during hospitalization. The antibiotics treatment was not indicated for acute diarrhea routinely. However, antibiotics were given for several circumstances, including suspected cases of septicemia or cholera, cases of bloody diarrhea, or diarrhea associated with another acute infection. Up to two-thirds of patients were prescribed antibiotics treatment. Amoxicillin was the most commonly prescribed antibiotic (52.3%), followed by ceftriaxone (38.0%). While there are no differences regarding the antibiotic prescription between the dehydration group and the non-dehydration one, a higher proportion of patients with dehydration was indicated to receive intravenous fluid compared to the non-dehydration group (93.8% vs. 41.2%, p = 0.001) (Table 3). All participants except two recovered on discharge. The remaining two patients were transferred to hospitals in Thailand according to their parents’ desire. The median duration of hospitalization was three days (range 1–7).

**Table 3:** Treatment and outcome of acute diarrhea among hospitalized U5 children.

	Total (n = 33)	No dehydration (n = 17)	Dehydration (n = 16)	p-value
**Treatment after admission**				
ORS	33 (100.0)	17 (100)	16 (100)	1.0*
IV fluid	22 (66.7)	7 (41.2)	15 (93.8)	**0.001** **
Antibiotics	22 (66.7)	11 (64.7)	11 (68.8)	0.8**
**Outcomes on discharge**				
Recovery	31 (93.9)	17 (100)	14 (87.5)	0.2*
Refer	2 (6.0)	0 (0.0)	2 (12.5)	
Death	0 (0.0)	0 (0.0)	0 (0.0)	
**Duration of hospitalization (days)**	3 (1–7)	2 (1–4)	3 (1–7)	0.054***

ORS: oral rehydration solution; IV: intravenous. Numbers in the parentheses indicate column percentage (%), unless indicated range for continuous variables.

*P-value was calculated by Fisher’s exact test.

**P-value was calculated by Chi-squared test.

***P-value was calculated by Mann-Whitney test.

### Association between etiologic pathogens and level of dehydration among U5 children hospitalized with acute diarrhea

There is a significantly higher prevalence of dehydration among children with acute diarrhea caused by rotavirus compared to those with negative rotavirus testing (70.0% vs. 12.5%, p = 0.02) (Table 4).

**Table 4:** Association between etiologic pathogens and level of dehydration among U5 children hospitalized with acute diarrhea.

	Rotavirus (n = 18)		p-value*	*Salmonella spp*. (n = 33)		p-value*	*Shigella spp*. (n = 33)		p-value*
	Negative (n = 8)	Positive (n = 10)		Negative (n = 29)	Positive (n = 4)		Negative (n-32)	Positive (n = 1)	
No dehydration	7 (87.5)	3 (30.0)	**0.02**	15 (51.8)	2 (50.0)	1.0	16 (50.0)	1 (100.0)	1.0
Dehydration	1 (12.5)	7 (70.0)		14 (48.2)	2 (50.0)		16 (50.0)	0 (0.0)	

*P-value was calculated by Fisher’s exact test. Numbers in the parentheses indicate column percentage (%).

## Discussion

In this study, the hospitalized diarrheal patients were predominantly boys and accounted for three-quarters. There is emerging evidence showing that boys are more affected by acute diarrhea compared with girls [10]. This gender-based difference was suggested to be the result of a cultural healthcare-seeking bias towards boys, differences in environmental exposures, and pathophysiology mechanisms between boys and girls [11, 12]. Among all participants, children of 0–35 months (infants and toddlers) were mostly affected with acute diarrhea. This finding is in line with previous studies showing that the prevalence of diarrheal illness gradually decreased as the children grew older [13, 14]. The two most common symptoms found in the pediatric patients admitted with acute diarrhea were vomiting and fever, similar to studies from Peru, Indonesia, and China [15–17]. These two symptoms could increase the level of dehydration and interfere with children’s feeding. If the children do not tolerate oral rehydration, enteral or IV rehydration may be needed [18]. Therefore, two-thirds of the participants received IV fluid even though some did not show signs of dehydration.

The isolated etiologic agents in this study were rotavirus, *Salmonella spp*., and *Shigella spp*. Due to the lack of laboratory equipment, the other viral infections and rotavirus genotypes could not be investigated. Although the testing for rotavirus could not be performed for all 33 cases, it showed a high prevalence of rotavirus infection among these children (55.5%). This finding is similar to the study of Souk Aloun *et al*, which reported that 55.0% of hospitalized U5 children with acute diarrhea in Vientiane, Lao PDR tested positive for rotavirus [19]. The data from other Asian countries also indicated that rotavirus was the leading cause of acute gastroenteritis among U5 children [20]. Although there is a high prevalence of rotavirus enteritis among U5 children, the rotavirus vaccination has not been introduced in Lao PDR. Recent studies suggested the good cost-effectiveness of the introduction of rotavirus vaccine in improving child health and reducing the economic burden of rotavirus enteritis in childhood [21, 22]. In the present study, the prevalence of bacterial infection was 15.1%, which is lower than other reports from low- and middle-income countries such as Burkina Faso and Bangladesh (40.0% and 39.2%, respectively) [23, 24]. This discrepancy may be because some patients in our study had received antibiotics before collecting the stool samples. All five cases with positive stool testing for pathogenic bacteria were not treated with antibiotics before the stool collection. The most prevalent bacterial pathogen was *Salmonella spp*. accounted for 80.0% (4 of 5 cases), similar to a study in China that found that *Salmonella spp*. was the leading cause of bacterial infection in U5 children with acute diarrhea [17]. Our study did not find a co-infection or combination of pathogens, which could be resulted from the long-distance transportation or stool collection technique to send the stool samples from Savannakhet Provincial Hospital to NCLE.

The main finding of this study was that children with acute diarrhea caused by rotavirus had a higher prevalence of dehydration compared with those with negative rotavirus testing, which is similar to several previous studies [16, 19, 25]. Although children with dehydration received more IV fluid than children without dehydration, there was no statistically significant association between positive rotavirus and receiving IV fluid. It might be due to the small number of cases. Eight out of ten rotavirus-positive cases and four out of eight negative cases received IV fluid.

This study has several limitations. First, due to the poor storage system of the hospital, only available data of 2% of U5 children hospitalized with acute diarrhea were analyzed. As a result, missing data bias might affect the study results. Second, the clinical records of the SPH failed to provide critical information for all hospitalized patients, such as nutrition status and breastfeeding. In the absence of a standardized recording system, the level of dehydration was not fully classified. Third, due to the limitation of the laboratory in SPH, all stool samples needed to be sent to NCLE, and the results were only sent back after a few weeks when the patients had already been discharged. As a result, not all pediatric patients hospitalized with acute diarrhea were indicated to collect their stool samples but only patients with moderate or severe dehydration. This is why we could only retrospectively report data of 33 patients during a 2-year study period. There should be further prospective studies to evaluate the viral and bacterial etiology of acute diarrhea among U5 children in Lao PDR in the future.

## Conclusions

In conclusion, our study showed that rotavirus was the most prevalent pathogen of acute diarrhea among U5 children. Pediatric patients with acute diarrhea caused by rotavirus had a higher prevalence of dehydration compared to those with negative rotavirus testing.

## Supporting information

S1 File (XLS)
